# Immune cell infiltrates in peritoneal metastases from colorectal cancer

**DOI:** 10.3389/fimmu.2024.1347900

**Published:** 2024-02-07

**Authors:** Patrik Sundström, Stephen Hogg, Marianne Quiding Järbrink, Elinor Bexe Lindskog

**Affiliations:** ^1^Department of Microbiology and Immunology, Institute of Biomedicine, University of Gothenburg, Gothenburg, Sweden; ^2^Department of Surgery, Institute of Clinical Sciences, Sahlgrenska Academy, University of Gothenburg, Gothenburg, Sweden; ^3^Department of Surgery, Region Västra Götaland, Sahlgrenska University Hospital, Gothenburg, Sweden

**Keywords:** peritoneal carcinomatosis, colorectal cancer, T cell, PD-1, IFN-γ, immune therapy

## Abstract

**Background:**

The presence of peritoneal metastases (PMs) in patients with colorectal cancer (CRC) confers a poor prognosis and only a minority of patients will benefit from the available treatment options. In primary CRC tumors, it is well established that a high infiltration of CD8^+^ effector T cells correlates to a favorable patient outcome. In contrast, the immune response induced in PMs from CRC and how it relates to patient survival is still unknown. In this study, we characterized the immune infiltrates and the distribution of immune checkpoint receptors on T cells from PMs from CRC, in order to evaluate the potential benefit of checkpoint blockade immunotherapy for this patient group.

**Methods:**

Surgically resected PM tissue from CRC patients (*n*=22) and synchronous primary tumors (n=8) were processed fresh to single cell suspensions using enzymatic digestion. Surface markers and cytokine production were analyzed using flow cytometry.

**Results:**

T cells dominated the leukocyte infiltrate in the PM specimens analyzed, followed by monocytes and B cells. Comparing two different PMs from the same patient usually showed a similar distribution of immune cells in both samples. The T cell infiltrate was characterized by an activated phenotype and markers of exhaustion were enriched compared with matched circulating T cells, in particular the checkpoint receptors PD-1 and TIGIT. In functional assays most cytotoxic and helper T cells produced INF-γ and TNF following polyclonal stimulation, while few produced IL-17, indicating a dominance of Th1-type responses in the microenvironment of PMs.

**Conclusion:**

Immune cells were present in all PMs from CRC examined. Although infiltrating T cells express markers of exhaustion, they produce Th1-type cytokines when stimulated. These results indicate the possibility to augment tumor-specific immune responses within PMs using checkpoint blockade inhibitors.

## 1 Introduction

Worldwide, colorectal cancer (CRC) is one of the most common malignant tumors and one of the greatest contributors to overall cancer mortality (1). Primary tumors can be treated with surgery alone or in combination with chemotherapy and radiotherapy. For metastatic disease, however, treatment options are limited, and the five-year survival is only 15%, regardless of treatment regimen (2). The most common metastatic site is the liver, followed by lungs and peritoneum, respectively. Peritoneal metastases (PMs) are found in approximately 10% of all cases of CRC (3). Patients with PMs from primary colorectal tumors that are eligible for attempted curative treatment often receive cytoreductive surgery (CRS) combined with hyperthermic intraperitoneal chemotherapy (HIPEC). Recently, however, a large randomized multicenter study indicated that Oxaliplatin based HIPEC did not offer benefit to the patient compared with CRS alone when patients were stratified by peritoneal cancer index (PCI) score (4). These negative results further emphasize the urgent need for new treatment options for these patients.

It is now generally accepted that tumor-infiltrating T cells contribute to anti-tumor immunity and a beneficial patient outcome in many tumor types, including CRC (5). In brain metastases from CRC, T cell infiltration is usually lower than in the primary tumors, while lung and liver metastases typically harbor more T cells than the primary tumor (6, 7). As in primary CRC tumors, degree of T cell infiltration into metastases in general correlates to better patient outcomes (6, 8). However, specific data on immune cell infiltration in PMs from CRC remain relatively scarce. Data so far indicates that there is a consistently lower T cell infiltration in PMs than in the primary tumor, especially at the invasive margin (9). It has also been shown that patients with PMs have increased frequencies of CD4^+^ memory T cells which express the checkpoint receptors PD-1, VISTA, and TIGIT in the omental fat when compared to patients with only a primary CRC tumor (10).

A recent study presented convincing evidence that CRC tumors giving rise to PMs predominantly belong to the consensus molecular subgroup (CMS) 4 subgroup, have increased KRAS pathway activation, and are microsatellite stable (MSS) (11). The PMs retained many of the features of the primary tumor and could be further subdivided into three subgroups with distinct molecular and clinical features (11). One of these subgroups was characterized by a relatively high immune cell infiltration and may be particularly interesting for future immune checkpoint immunotherapy (ICI).

ICI has emerged as a potent antitumor treatment for several tumors. In CRC, however, ICI therapy benefits only the relatively small subset of patients with high microsatellite instability (MSI-H) tumors (12, 13). These tumors typically demonstrate a greater influx of T cells and carry a much higher tumor mutational load. To investigate the potential of ICI treatment in the subset of patients with PM from CRC, we characterized the immune cell infiltrate in PMs along with the activation and exhaustion states of the T cells present, and compare with the patient’s blood and, where possible, primary tumor. Our results demonstrate that T cells dominate the leukocyte infiltrate of PMs from CRC and that CD8^+^ T cells in PMs have an activated phenotype, produce type 1 cytokines, and express several exhaustion markers. Furthermore, the expression of PD-1 ligands by myeloid cells in a subset of PMs lend weight to the argument that ICI might be beneficial for some CRC patients with peritoneal metastasis.

## 2 Materials and methods

### 2.1 Patients and tissue collection

Consecutive patients planned for CRS/HIPEC at Sahlgrenska University Hospital for colorectal cancer, comprising adenocarcinoma in the appendix, were included in the study. An informed written consent was obtained from all patients and the study was conducted according to the declaration of Helsinki and was approved by the Swedish Ethical Review Authority (no. 543-17). The tissue material was collected during surgery and transported in ice-cold PBS before isolation of lymphocytes within two hours. Heparinized venous blood was also obtained during surgery. Information about tumor stage was retrieved from the pathology report and medical records. Microsatellite instability, indicating the mutational load of the tumor, was analyzed as previously described (14) or retrieved from the pathology report.

### 2.2 Cell isolation and stimulation

PBMC were isolated by gradient centrifugation on Ficoll-Paque™Plus (GE Healthcare Bio-sciences AB). Tumor-infiltrating cells from primary tumors were isolated as previously described (15). In PMs, tumor-infiltrating lymphocytes were isolated employing the same enzymes and incubation times. Briefly, the tissue was cut into 5 mm pieces and washed twice for 15 minutes at 37°C in RPMI 1640 (GIBCO^®^ by Life Technologies™) containing 10% fetal bovine serum (SIGMA-ALDRICH^®^), 1mM of hepes, 1mM of sodium pyruvate, 50 μM 2-mercaptoethanol, 100 U/ml of penicillin, 100 μg/ml of streptomycin, 292 μg/ml of L-glutamine (GIBCO™) during gentle stirring. Tissue digestion was then performed using Liberase™ and DNase I (Roche) in the same medium, supplemented with 5µM CaCl_2_, and stirred gently for 1.5-2 hours. The resulting single cell solutions were re-suspended in RPMI 1640 (GIBCO^®^ by Life Technologies™) containing 10% fetal bovine serum (SIGMA-ALDRICH^®^), 25mM of hepes, 100 U/ml of penicillin, 100 μg/ml of streptomycin, 292 μg/ml of L-glutamine (GIBCO™), and 50 μg/ml of gentamicin (GIBCO™). Cells were counted using a flow cytometry panel with antibodies to CD3, CD4, CD8, and CD45 and Fixable Viability Stain (see **Supplementary Table 1** for antibodies used for flow cytometry) and analyzed on a Beckman Coulter Cytoflex cytometer. For phenotypic analyses, staining was performed directly after isolation. For cytokine production analyses, cells were incubated overnight in culture medium at 37°C before polyclonal stimulation the following morning using 50ng/mL of phorbol 12-myristate 13-acetate (PMA) and 680ng/mL of ionomycin calcium salt (Sigma Aldrich) for 4 hours, together with a protein transport inhibitor (BD Golgi stop, BD Biosciences).

### 2.3 Flow cytometry analyses

Single cell suspensions were stained with antibodies to surface markers (see **Supplementary Table 1** for antibodies used for flow cytometry). eBioscience™ Foxp3/Transcription Factor Staining Buffer Set (Invitrogen) was then used for detection of Ki67, Foxp3 and cytokines. Viable lymphocytes were identified by their forward and side scatter characteristics combined with staining for CD45, and Fixable Viability Stain 575V or 510 (both from BD Horizon™). Fluorescence minus one controls were used to determine positive staining of surface markers and unstimulated cells to determine positive cytokine staining. Data was acquired using a Becton Dickinson Fortessa X-20 flow cytometer and analyzed by FlowJo v10 software. A cut-off minimum of 100 events in a certain cell population was applied in all analyses of cell surface markers and effector molecules.

### 2.4 Statistical methods

Statistical analyses of paired data were performed using two-tailed Wilcoxon matched-pairs signed rank test. Statistical significance was determined using p<0.05.

## 3 Results

### 3.1 Patient characteristics

All 22 patients included in the study were planned for CRS/HIPEC due to PMs arising from colorectal cancer. The median age of the patients was 62 years, 12 patients were male and 10 were female. Of the 22 patients, CRS/HIPEC was performed in 16 patients, CRS alone in 2, while in four patients, disease was too advanced, and the approach changed to open-close surgery. Patient and tumor characteristics are presented in **Table 1**.

**Table 1:** Patient and primary tumor characteristics.

**Age**, median *(range*)	62	(31–73)
Gender, *n* (%)		
Male	12	(55)
Female	10	(45)
Primary tumor location, *n* (%)*		
Colon, right sided	10	(45)
Appendix	4	(18)
Colon, left sided	7	(32)
Rectum	1	(5)
Peritoneal metastases, *n* (%)		
Synchronous	13	(59)
Metachronous	9	(41)
Tumor stage primary tumor, *n* (%)		
pT3	3	(14)
pT4	17	(77)
Missing^†^	2	(9)
Node stage primary tumor, *n* (%)*		
pN0	1	(5)
pN1	7	(32)
pN2	12	(55)
Missing^†^	2	(9)
Tumor differentiation, *n* (%)		
High grade^‡^	9	(41
Low grade	4	(18)
Mucinous	7	(32)
Missing^†^	2	(9)
Microsatellite stability, n (%)		
MSS	21	(95)
MSI-H	0	
Missing	1	(5)
**PCI score,** median	19	(5–34)

PCI, Peritoneal Cancer index. * Percentages do not add up due to rounding. ^†^ Two patients missing due to no primary resection. ^‡^One patient had goblet cell adenocarcinoma.

### 3.2 T cells dominate the leukocytes infiltrating peritoneal metastases.

**Figure 1:**
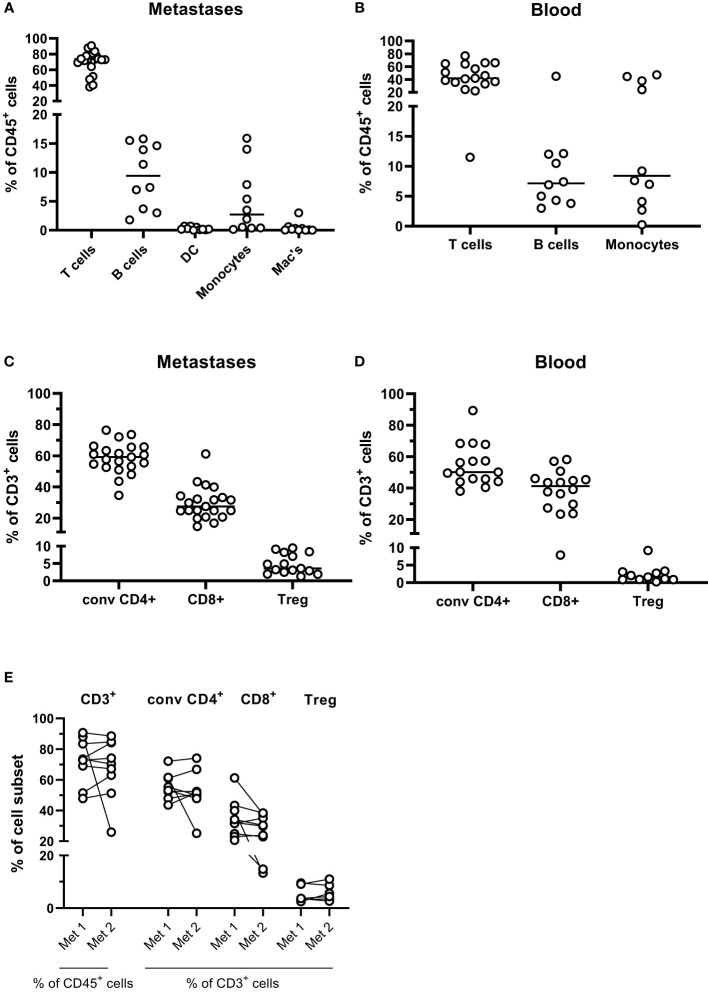
Leukocyte subsets in peritoneal metastases. Single cells suspensions were prepared from peritoneal metastases (PMs) and blood and analyzed by flow cytometry to determine the distribution of leukocyte subsets in PMs **(A)** and blood **(B)**. Distribution of conventional CD4^+^ and CD8^+^ T cells and regulatory T cells (Treg) was determined among all CD3+ T cells in PMs **(C)** and blood **(D)**. The T cell composition was also compared in cell suspensions from synchronous metastases **(E)**. Symbols represent individual patients, and in **(E)** the two metastases from individual patients are connected with lines. n=9-21.

To get a broad overview of immune cell infiltration into PMs from CRC, we first performed immune phenotyping of cells isolated from tumor tissues based on surface markers detected by flow cytometry. Manual gating was used to identify T cells (CD3^+^ cells), B cells (CD19^+^ cells), dendritic cells (CD3^-^CD19^-^CD14^-^CD11c^+^HLA-DR^+^ cells), monocytes (CD3^-^CD19^-^CD14^+^CD11c^+^ cells), and macrophages (CD3^-^CD19^-^CD14^+^CD11c^-^ cells) among CD45^+^ cells, after gating on forward/side scatter characteristics and viability (See **Supplementary Figure 1** for full gating strategy). Making an absolute distinction between monocytes and macrophages based on surface markers can be difficult; we chose to use the expression of CD11c as a marker for monocytes recently recruited from the circulation, while CD11c- cells represent macrophages maturing from recruited monocytes and stationary macrophages (16). These analyses demonstrate that T cells dominate the leukocyte infiltrate in the PMs, followed by B cells and monocytes (**Figure 1A**). Dendritic cells and macrophages were present in all the PMs, but in significantly lower numbers than monocytes. Comparing with blood from the same patient, T cells were clearly enriched in the PMs, while monocytes were less frequent than in the circulation (**Figures 1A, B**). None of the patients had MSI-H PMs and the effect of microsatellite instability on lymphocyte infiltration could thus not be evaluated.

T cells were present in all PM specimens but varied considerably in numbers (between 0.2x10^6^ and 17x10^6^ CD3^+^ cells/g tissue, median 4.9x10^6^). A separate flow cytometry panel was used to distinguish between CD8^+^ T cells, Treg (CD4^+^CD127^low^CD25^bright^FoxP3^+^) and conventional CD4^+^ non-Treg cells (See **Supplementary Figure 2** for full gating strategy). In PMs, conventional CD4^+^ T cells were more frequent than CD8^+^ T cells, while in the circulation the frequencies of CD4^+^ and CD8^+^ T cells were more similar (**Figures 1C, D**). Both conventional CD4^+^ T helper and CD8^+^ T cells are needed for an efficient anti-tumor immune response, while relative Treg enrichment generally correlates with poorer patient outcomes. Consistent with analyses of primary CRC tumors, Treg were significantly enriched in the metastases compared with the circulation (p<0.05; **Figures 1C, D**). Moreover the CD8^+^:Treg cell ratio was significantly lower in the metastases than in the circulation (median 8.2 in the metastases and 20.3 in the circulation, p<0.05; **Supplementary Figure 3**).

There was substantial inter-individual variation in both total leukocyte infiltration and the composition of the infiltrate in PMs. For nine patients, tissue from two synchronous metastases were available for analysis. Generally, the degree and composition of T cell infiltration in discrete PMs from a patient was more similar than between infiltrates in PMs from different individuals (**Figure 1E**). However, in two patients with metastases to small intestine, frequencies of T cells were significantly lower than synchronous PMs from other locations.

Comparison of T cell infiltration in primary tumors and PMs was possible in 4 patients. Of note, no significant differences were found between the composition of T cell infiltrates in primary tumors and associated metastases (**Supplementary Figure 4A**). However, the density of T cells in these tissues varied considerably (**Supplementary Figure 4B**).

In summary, we show that T cells and monocytes dominate among immune cells in PMs from CRC, and that the individual composition of immune cell subsets is relatively well preserved in different metastases from the same individual.

### 3.3 T cells in peritoneal metastases express markers of both activation and exhaustion

**Figure 2:**
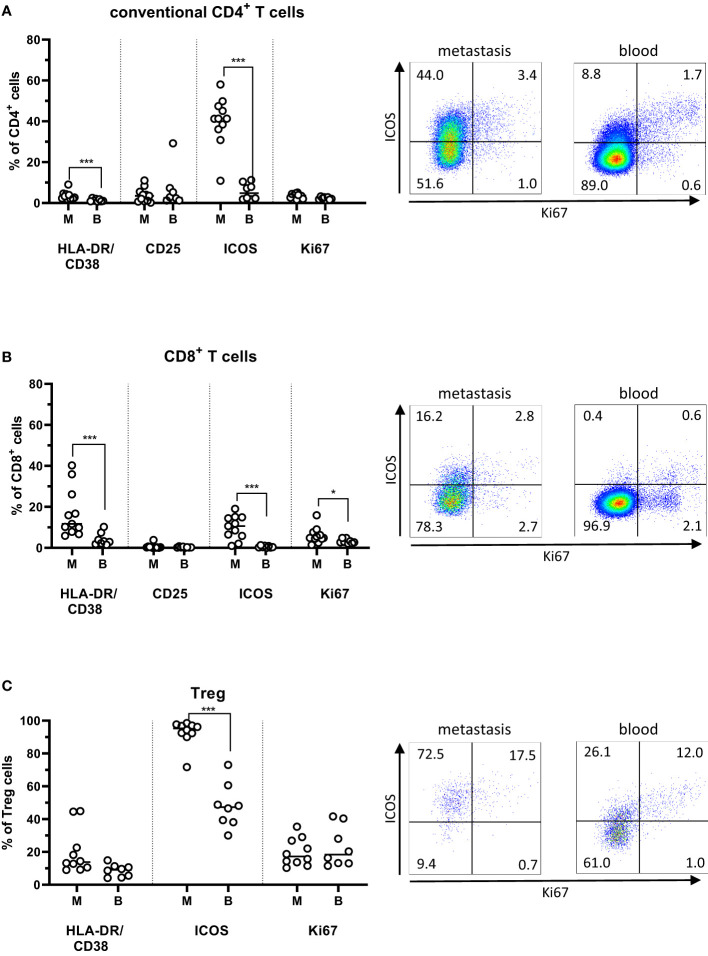
Activation markers on T cells from peritoneal metastases. Single cells suspensions were prepared from peritoneal metastases (PMs) and blood and analyzed by flow cytometry to determine the distribution of activation markers on conventional CD4^+^ T cells **(A)**, CD8^+^ T cells **(B)**, and regulatory T cells (Treg; **C**) in PMs and blood. Dot plots show representative staining of the respective cell subsets from PMs and blood. Symbols represent individual patients and lines the median. M – metastasis, B – blood, * p<0.05, *** p<0.001, n=8-11.

To assess T cell activation *in situ* in the PMs, we determined the combined expression of HLA-DR and CD38, as well as CD25 and ICOS (CD278). There were increased frequencies of HLA-DR^+^CD38^+^ activated conventional CD4^+^ T cells in the metastases compared to the circulation (p<0.001). In addition, CD8^+^ T cells in PMs also had a high co-expression of HLA-DR and CD38, which was actually higher than in both the metastasis-infiltrating CD4^+^ cells and circulating CD8^+^ cells (p<0.001; **Figures 2A, B**). Moreover, ICOS expression was considerably higher in tumor-infiltrating T cells than circulating T cells, particularly among conventional CD4^+^ T cells (p<0.001). *In situ* proliferation was determined by expression of Ki67. Of note, Ki67 expression was similar between tumor-infiltrating and circulating CD4^+^ T cells (**Figure 2A**) but was increased in CD8^+^ T cells in metastases compared to the circulation (p<0.05; **Figure 2B**).

In general, Treg in PMs exhibited a more activated phenotype and higher frequencies of Ki67 expression than the conventional CD4^+^ and CD8^+^ T cells in the same PMs. Additionally, ICOS expression was significantly higher among metastasis-infiltrating Treg than among circulating Treg (p<0,001; **Figure 2C**).

**Figure 3:**
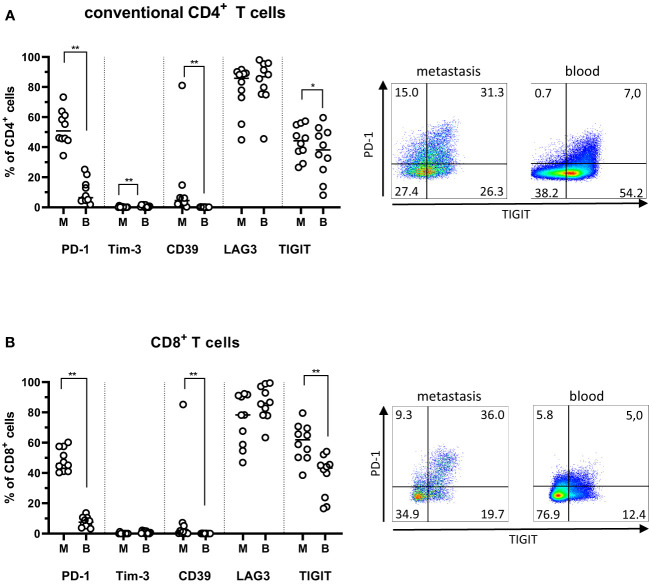
Exhaustion markers on T cells from peritoneal metastases. Single cells suspensions were prepared from peritoneal metastases (PMs) and blood and analyzed by flow cytometry to determine the distribution of exhaustion markers on conventional CD4^+^ T cells **(A)** and CD8^+^ T cells **(B)** in PMs and blood. Dot plots show representative staining of the respective cell subsets from PMs and blood. Symbols represent individual patients and lines the median. M – metastasis, B – blood, * p<0.05, ** p<0.01, n=10.

Several of the most studied markers of exhaustion, including PD-1 and Tim-3, are also expressed on recently activated T cells, meaning that it can be difficult to unequivocally distinguish between activation or exhaustion. In the tumor microenvironment, however, it is generally agreed that these markers primarily identify exhausted cells (17, 18). In PMs, there was a substantial expression of PD-1, LAG3, and TIGIT by both the conventional CD4^+^ and CD8^+^ T cells (**Figures 3A, B**). LAG3 expression was particularly high, and this was also the case among circulating T cells. In contrast, PD-1- and TIGIT-expressing T cells were significantly more frequent in the PMs than circulation (P<0.01; **Figure 3B**). Even though frequencies of CD39^+^ T cells were much lower than frequencies of PD-1- or TIGIT-expressing T cells, they were still significantly higher in PMs than among matched circulating T cells (p<0.01). Approximately half the CD39^+^ T cells in PMs co-expressed PD-1 (**Supplementary Figure 5**), indicating that they may be terminally exhausted cells specific for tumor antigens (19, 20). Again, with regard to expression of exhaustion markers, there was concordance between synchronous metastases from single individuals (**Supplementary Figure 6).**

Taken together, these data show that frequencies of exhausted T cells are increased in the tumor microenvironment of PMs compared to blood; in a similar vein, there appears to be a significant population of activated T cells in PMs, indicating the possibility to generate tumor-specific immune responses.

### 3.4 PD-1 ligands in metastases are primarily expressed on myeloid immune cells

**Figure 4:**
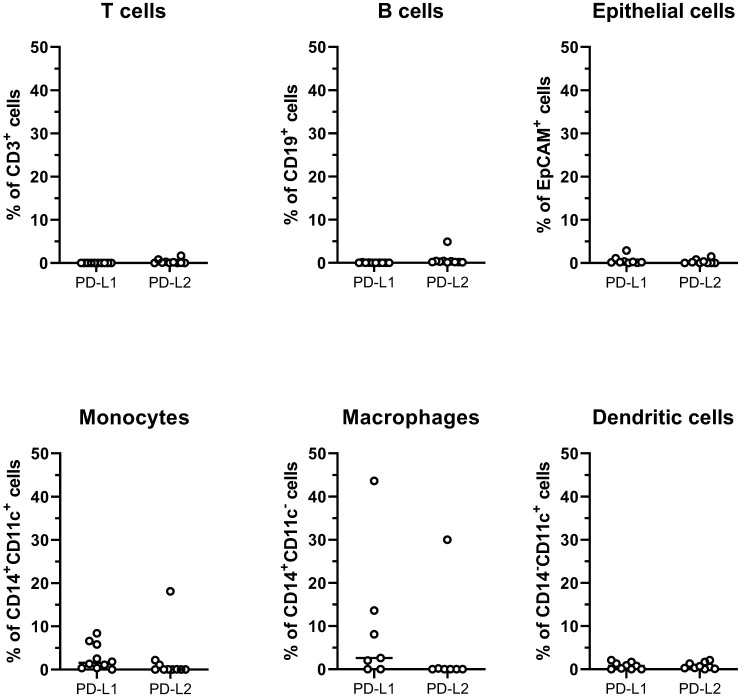
PD-1 ligands on cells from peritoneal metastases. Single cells suspensions were prepared from peritoneal metastases (PMs) and analyzed by flow cytometry to determine the distribution of PD-L1 and PD-L2 on T cells, B cells, tumor epithelial cells, monocytes, macrophages, and dendritic cells. Symbols represent individual patients and lines the median. n=7-10.

To evaluate the possibility to improve tumor-specific immune responses by immune checkpoint-blockade, we determined expression of the two PD-1 ligands, PD-L1 and PD-L2 on immune cells and tumor epithelial cells (defined as CD45^-^EpCAM^+^ cells). It should be noted that the cell isolation protocol used has been optimized to generate lymphocytes; therefore, the yield of both myeloid cells and epithelial cells may well underrepresent true tissue numbers. Nonetheless, it is clear that PD-L1 and PD-L2 expression was very low on epithelial cells, B cells, and T cells (**Figure 4**). The highest expression was found on monocytes and macrophages, but the interindividual variation was large. Of note, PD-1 expression frequencies on CD4^+^ or CD8^+^ T cells did not correlate with ligand expression on monocytes or macrophages (data not shown).

### 3.5 Cytokine production by tumor-infiltrating T cells

**Figure 5:**
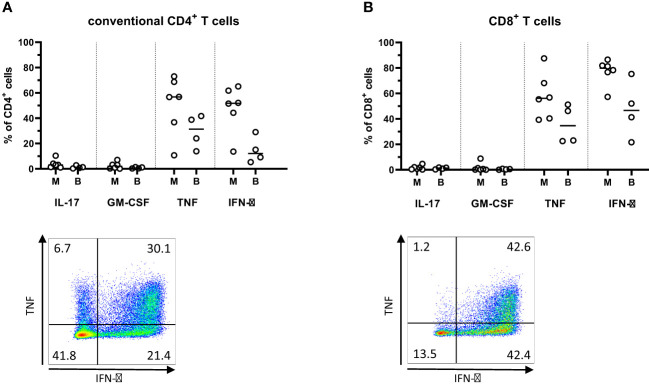
Cytokine production in T cells from peritoneal metastases. Single cells suspensions were prepared from peritoneal metastases (PMs) and blood, stimulated with PMA and ionomycin for four hours, and analyzed by flow cytometry to determine the production of cytokines by conventional CD4^+^ T cells **(A)** and CD8^+^ T cells **(B)** in PMs and blood. Dot plots show representative staining of the respective cell subsets from PMs. Symbols represent individual patients and lines the median. M – metastasis, B – blood, n=4-6.

In order to investigate the functional properties of PM infiltrating T cells, cytokine production was analyzed following polyclonal stimulation with PMA and Ionomycin. These analyzes showed a predominant production of Th1-type cytokines IFN-γ and TNF from T cells in all the analyzed tissues (**Figure 5**). In particular, the great majority of CD8^+^ T cells from the PMs produced IFN-γ. In contrast, the production of the Th17-associated tumor-promoting cytokines IL-17 and GM-CSF was comparatively lower in both the CD4^+^ and CD8^+^ T cells isolated from PMs. (**Figure 5**). Generally, the differences between synchronous metastases were larger when comparing cytokine production than when comparing expression of surface markers (**Supplementary Figures 7A, B**).

This is, to our knowledge, the first assessment of cytokine production in PMs from CRC, we sought to compare these analyses with a better-studied tissue. Therefore, we also analyzed cytokine production in T cells isolated from primary CRC tumors from six additional patients (4 with MSI-H tumors and 2 with MSS tumors). In these tumors, we also found low IL-17 and GM-CSF production, in parallel with higher IFN-γ production by T cells. Generally, Th1-type cytokine production by T cells was not found to the same extent as that seen in PMs, while IL-17- and GM-CSF-production in conventional T cells was somewhat higher than in the PMs (**Supplementary Figures 7C, D**).

We thus conclude that the T cell cytokine production in PMs is dominated by Th1 type cytokines, and that it is equivalent to, if not higher than, that detected in primary tumors.

## 4 Discussion

It is now generally accepted that tumor-infiltrating T cells contribute to anti-tumor immunity and a beneficial patient outcome. Indeed, this notion was first established in CRC (21) and has since evolved to generate both the immunoscore as a standardized method to evaluate the extent of a intratumoral anti-tumor immune response, and a detailed knowledge of the importance of different immune mediators and cell subsets (5, 22). In stark contrast to primary CRC tumors, there is little information about the immune response to PMs originating from CRC. In this study, we sought to characterize the immune cell infiltrate in PMs and could show that T cells are present in all metastases, and that these T cells constitute a diverse population of cells expressing markers of both activation and exhaustion.

The immune cells infiltrating PMs has so far only been characterized with regard to basic T cell markers by immunohistochemistry (IHC) (9) and showed a lower infiltration of CD4^+^, CD8^+^ and Treg in PMs compared to primary tumors. Furthermore, CD4^+^ T cells were more numerous than CD8^+^ cells in the tumor core of PMs, a finding that was confirmed in our study. A recent seminal report by Lenos et al. (11) demonstrated that the large majority of colorectal tumors giving rise to PMs represent the CMS4 subtype, and that the molecular characteristics of the primary tumors are maintained in the PMs. CMS4, or mesenchymal type, tumors are characterized by KRAS pathway mutations, TGF-β activation, immune suppression, and stromal invasion (23), and often these patients have poorer survival compared to the other CMS subtypes (24). Lenos et al. (11) further identified 3 clusters of CMS4 PMs, and showed that one cluster, comprising about half of the patients, had a high immune cell infiltration and immune signaling pathways - similar to those of MSI-H CRC. They also showed increased expression of checkpoint receptors in this cluster. These transcriptional data on immune reactions in PMs are further advanced with our cellular analyses of the immune cells infiltrating PMs.

When analyzing the local immune reactions in PMs from CRC, we found a substantial population of CD8^+^ T cells expressing markers of activation, with the ability to secrete IFN-γ and TNF upon stimulation. This indicates the potential to mount protective immune responses if properly stimulated. Extensive studies on immune responses in primary CRC tumors have demonstrated that infiltration of CD8^+^ effector T cells with cytotoxic potential and production of Th1 type cytokines correlates with a better prognosis (25, 26). However, our study demonstrated that a high proportion of PM-infiltrating CD8^+^ T cells also expressed the checkpoint receptors PD-1 and TIGIT, indicating exhaustion. Exhaustion is characterized as a reversible state of sequential loss of effector functions, mediated by signaling through one or several checkpoint receptors (17, 18). We could also demonstrate expression of the PD-1 ligands PD-L1 and PD-L2 on monocytes and macrophages in the PMs, indicating an active maintenance of exhaustion in the PM microenvironment. This observation is in line with previous findings in primary CRC, where PD-1 ligand expression is primarily detected on hematopoietic cells, and PD-L1 expression on tumor cells is low (27–29).

Interestingly, a fraction of the putative exhausted cells in both the CD4^+^ and CD8^+^ subsets also expressed CD39. Co-expression of PD-1 and CD39 have previously been identified as specific for a T cell subset of tumor-specific T cells (30–32), potentially able to generate tumor-specific immune responses upon ICI. Expression of Ki67 by these cells may also indicate an active anti-tumor response. In non-small cell lung cancer patients, circulating Ki67^+^CD8^+^ T cells expressing PD-1 were seen following ICI, primarily in the responding individuals (33). A similar increase in proliferating CD8^+^ T cells with an “exhausted” phenotype was also seen in patients with malignant melanoma after ICI treatment (34, 35). Indeed, in keeping with our observations in PMs, the proliferating CD8^+^ cells found in ICI-responsive lung cancer patients co-expressed PD-1, HLA-DR and CD38. Furthermore, a recent study showed that a high CD3^+^ to CD4^+^ ratio in PMs from CRC, i.e. a dominance of CD8^+^ T cells over conventional CD4^+^ T helper cells and Treg, correlated with better overall survival following cytoreductive surgery and HIPEC (9). Taken together, these observations indicate that PM patients with a high proportion of proliferating CD8^+^ T cells may be benefit from ICI.

However, the final outcome of the local immune response is formed by the balance between stimulating and immunoregulatory signals in the tumor microenvironment. One component potentially contributing to suppression of anti-tumor immunity in PMs is the infiltration of Treg. It is well documented that Treg accumulate in primary colorectal tumors compared to blood and unaffected colon (36–38), and this was also shown in a recent IHC-based study of PMs (9). Using flow cytometry, we can now confirm a similar enrichment of Treg in PMs from CRC. The proportion of CD4^+^ cells defined as Treg was higher in the IHC study (9), but this may be explained by the more rigorous identification of Treg by flow cytometry, where they are defined as CD25^bright^CD127^low^FoxP3^+^ cells, as compared to FoxP3 alone in IHC. In primary tumors, effector Treg confer a poor prognosis (36) and the expression of CD39 (data not shown), HLA-DR, CD38, and ICOS on Treg in PMs suggests that they are activated effector Treg (37, 39) and may have similar effects. Thus, there is both an increased frequency of Treg in PMs and a phenotype suggesting potent suppressive ability. In addition to Treg, the presence of Th17 type responses may also contribute to a tumor-promoting microenvironment and an unfavorable patient outcome (25, 26, 40, 41). Th17 cells promote angiogenesis, epithelial growth, and recruitment of myeloid cells, all supporting tumor progression (42). Here, however, we were unable to demonstrate any sizable Th17 responses in the PMs, suggesting that this pathway is not one of the major contributors to tumor immune evasion in peritoneal metastases from CRC.

There was a relatively high variation between patients in number of tumor-infiltrating T cells and their expression of the examined activation and exhaustion markers in the PMs. This may be explained by the presence of distinct clusters within the predominant CMS4 subtype PMs (11). Despite this interindividual variation, it is notable that the T cell composition was similar between synchronous PMs from the same patient, as was the expression of exhaustion markers. This is in keeping with previous findings of a preserved transcriptional profile between multiple PMs from one tumor (11), and contrasts to other metastatic sites (liver, lungs, brain), where the immune cell infiltrate varies considerably when compared to the primary tumor and between synchronous metastases (6–8). This difference may be explained by the special requirements of tumor cells metastasizing to the peritoneal cavity, for example the ability to survive as free-floating cells and to adhere to and invade organs from the outside rather than through transendothelial invasion, and the default immunoregulatory/anti-inflammatory environment of the omentum and peritoneal cavity in steady state, reducing the immune pressure during metastasis development (43). In any event, the analyses of PMs indicate that significant information about both tumor type and infiltrating immune cells may be available by sampling a single peritoneal metastatic site.

This is a single center study with a well-defined patient cohort. However, a limitation of the study is the relatively small number of patients included. The variation between tumors in stage and PCI score may have contributed to some of the variation seen between tumors. With a larger patient cohort, we may have been able to detect differences in the immune response between these groups. In this study, we have focused primarily on T cells, and this may be another potential limitation as the relative contributions of other leukocytes in anti-tumor immunity in the context of PM fall outside the scope of this work.

Taken together, this study demonstrates a consistent infiltration of T cells in PMs from CRC, and these T cells proliferate and express markers of both activation and exhaustion. In spite of some inter-individual variability, these findings indicate that CRC patients with PMs may benefit from ICI. The subgroup most likely to benefit may be those patients with higher degrees of immune cell infiltration and higher infiltrates of T cells expressing immune checkpoint receptors.

## Data availability statement

The raw data supporting the conclusions of this article will be made available by the authors, without undue reservation.

## Ethics statement

The studies involving humans were approved by Swedish Ethical Review Authority. The studies were conducted in accordance with the local legislation and institutional requirements. The participants provided their written informed consent to participate in this study.

## Author contributions

PS: Investigation, Methodology, Project administration, Writing – review & editing. SH: Investigation, Methodology, Writing – review & editing. MQJ: Conceptualization, Formal Analysis, Funding acquisition, Supervision, Writing – original draft, Writing – review & editing. EBL: Conceptualization, Formal Analysis, Investigation, Resources, Writing – review & editing. Funding acquisition.
