# Supplementary material for: Immune cell infiltrates in peritoneal metastases from colorectal cancer
Source: Front Immunol. 2024 Feb 7;15:1347900. doi: 10.3389/fimmu.2024.1347900 (PMC10879551; doi:10.3389/fimmu.2024.1347900)
Supplement: Supplementary file 1 [file Presentation_1.pdf]

Supplementary Material

Immune Cell infiltrates in Peritoneal Metastases from Colorectal Cancer

Patrik Sundström, Stephen Hogg, Marianne Quiding Järbrink*, Elinor Bexe Lindskog

*** Correspondence:** Marianne Quiding Järbrink: marianne.quiding@microbio.gu.se

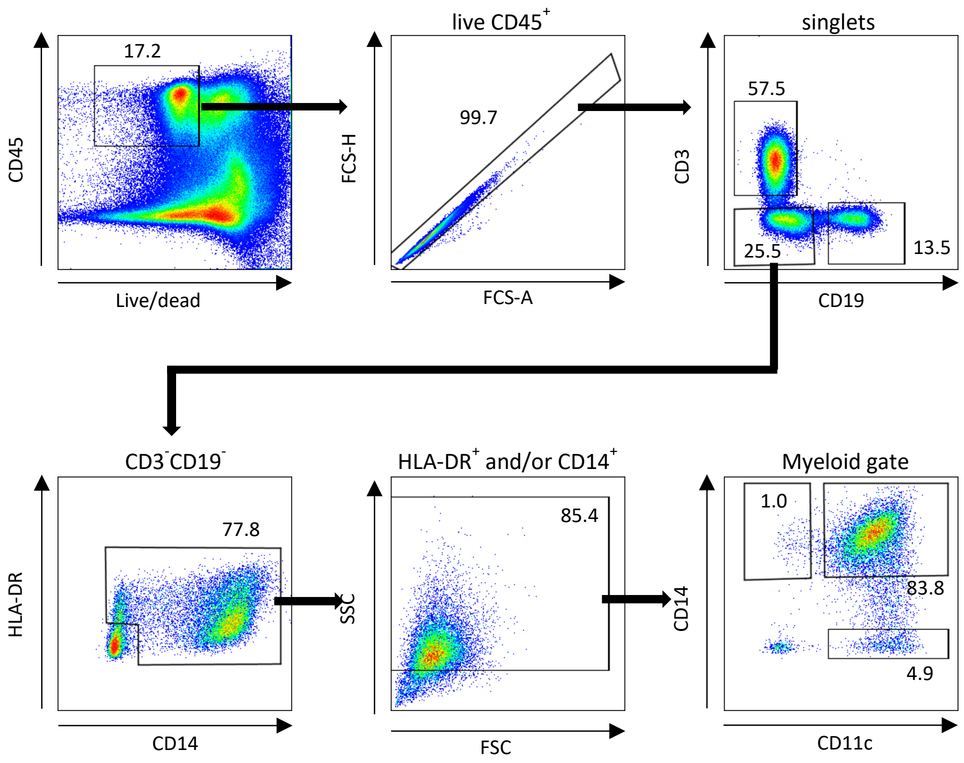

Suppl. Fig. S1. Gating strategy to identify T cells, B cells, monocytes, macrophages, and dendritic cells. One representative sample from a peritoneal metastasis is shown.

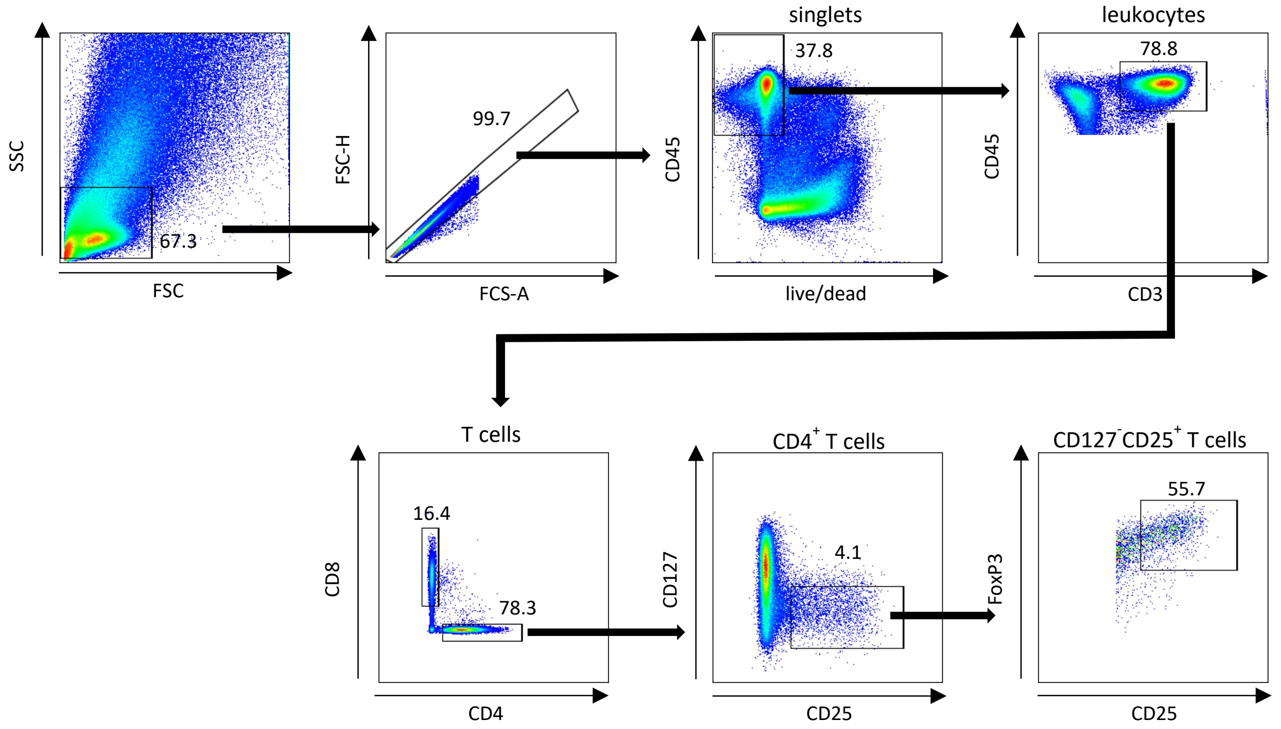

Suppl. Fig. S2. Gating strategy to identify CD8$^+$ T cells, conventional CD4$^+$ T cells and regulatory T cells. One representative sample from a peritoneal metastasis is shown.

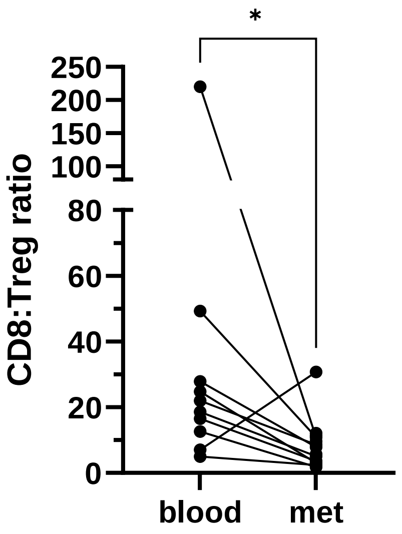

Suppl. Fig. S3. $CD8^+$ to regulatory T cell (Treg) ratio in peritoneal metastases. Single cells suspensions were prepared from peritoneal metastases (PMs) and blood and analyzed by flow cytometry to determine the frequencies of $CD8^+$ T cells and Treg in PMs and blood. The graph shows the ration of $CD8^+$ T cells to Treg. Symbols represent individual patients and the data points from each patient are connected with lines. * $p<0.05$, $n=10$

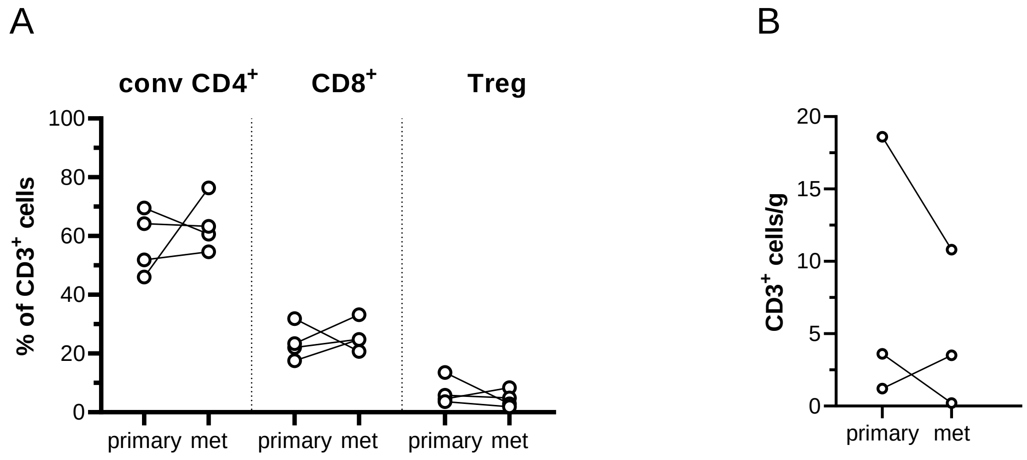

Suppl. Fig. S4. T cell frequencies in peritoneal metastases and primary tumors. Single cells suspensions were prepared from peritoneal metastases and synchronous primary tumors and analyzed by flow cytometry to determine (A) the frequencies of conventional $CD4^+$ T cells, $CD8^+$ T cells and regulatory T cells (Treg) and (B) the number of T cells per gram of tissue. Symbols represent individual patients and the data points from each patient are connected with lines. n=3-4

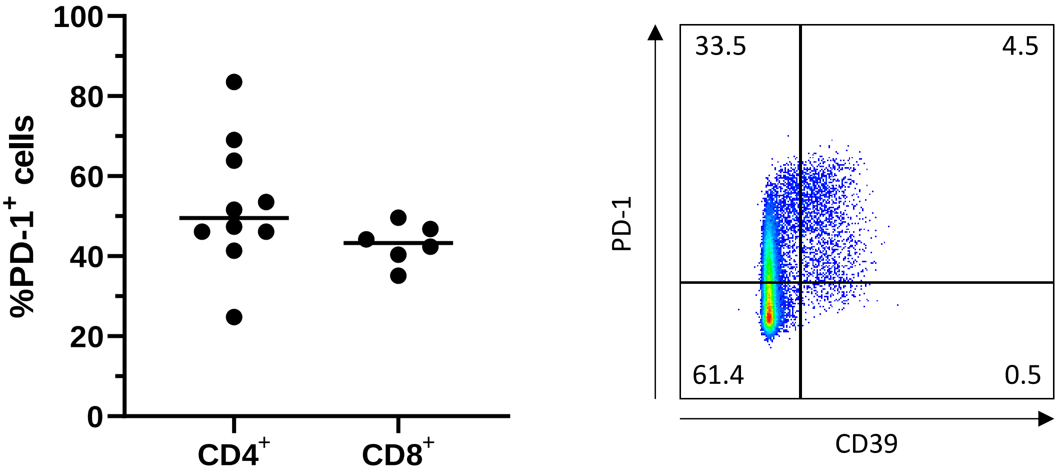

Suppl. Fig. S5. PD-1 and CD39 co-expression by T cells in peritoneal metastases. Single cells suspensions were prepared from peritoneal metastases and analyzed by flow cytometry to determine the frequencies of PD-1-expressing cells among CD39$^+$CD4$^+$ and CD39$^+$CD8$^+$ T cells. Symbols represent individual patients, and the dot plot shows a representative staining of CD8$^+$ cells. n=6-10

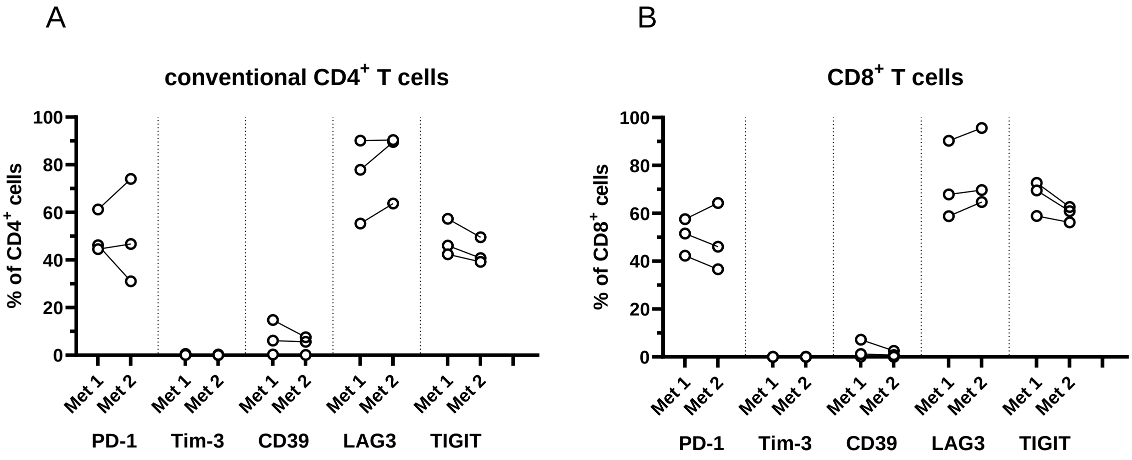

Suppl. Fig. S6. Exhaustion markers on T cells from peritoneal metastases. Single cells suspensions were prepared from peritoneal metastases (PMs) and analyzed by flow cytometry to determine the distribution of exhaustion markers on conventional $CD4^+$ T cells (A) and $CD8^+$ T cells (B) from synchronous PMs. Symbols represent individual patients, and the two metastases from individual patients are connected with lines. n=3

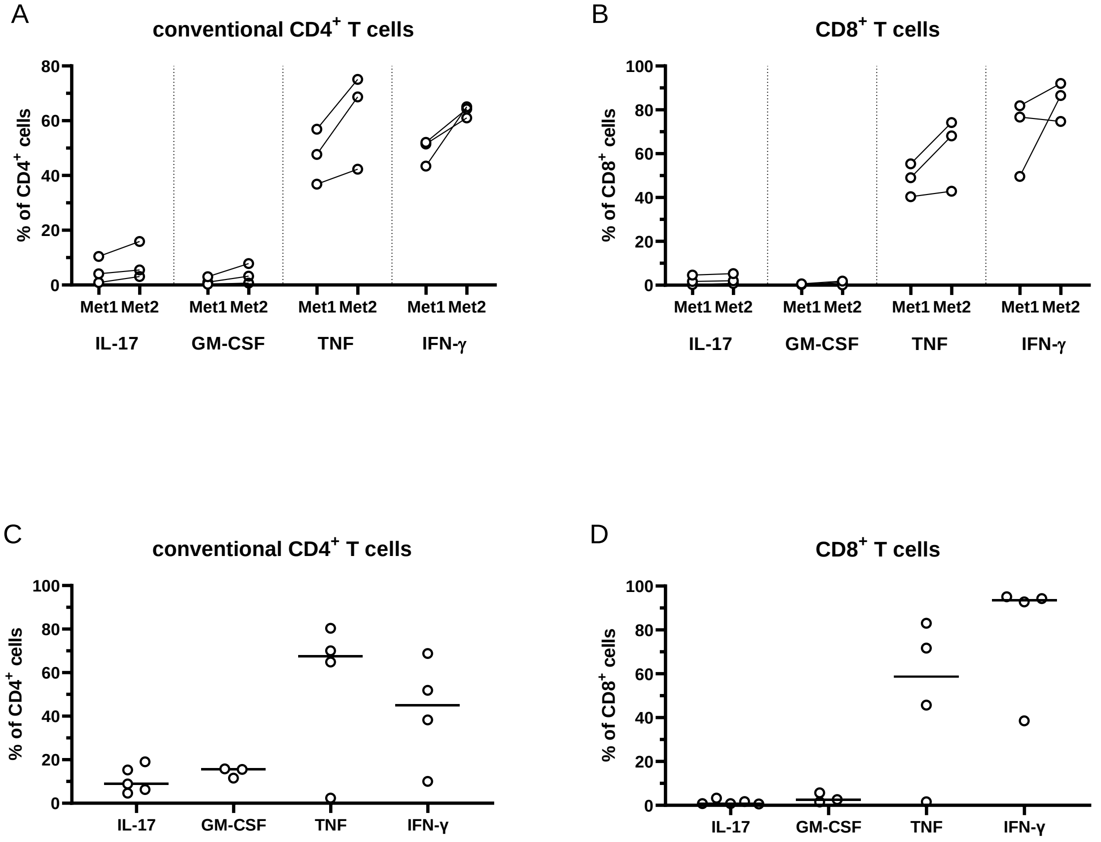

Suppl. Fig. S7. Cytokine production in T cells from peritoneal metastases. Single cells suspensions were prepared from synchronous peritoneal metastases (PMs), stimulated with PMA and ionomycin for four hours, and analyzed by flow cytometry to determine the production of cytokines by conventional $CD4^+$ T cells (A) and $CD8^+$ T cells (B). Single cells suspensions from primary colorectal tumors were stimulated and in the same way to determine the production of cytokines by conventional $CD4^+$ T cells (C) and $CD8^+$ T cells (D). Symbols represent individual patients, and in (A) and (B), the two metastases from individual patients are connected with lines. n=3-5

Supplementary table S1. Antibodies used for flow cytometry analyses.

**marker**	**Ab clone**	**fluorochrome**	**distributer**
CD3	SK7	APC-H7	BD Biosciences
CD4	OKT4	FITC, APC, PE/Dazzle	BioLegend
CD8	RPA-T8	BUV395	BD Biosciences
CD11c	B-ly6	PE-Cy7	BD Biosciences
CD14	MφP9	BV421	BioLegend
CD19	SJ25C1	PE	BD Biosciences
CD25	M-A251	BV650	BD Biosciences
CD38	HIT2	PerCP-Cy5.5	BD Biosciences
CD39	A1	FITC	BioLegend
CD45	2D1	AF-700	BD Biosciences
CD69	FN50	APC	BD Biosciences
CD127	hIL-7R-M21	PE	BD Biosciences
EpCAM	9C4	BV605	BioLegend
FoxP3	PCH101	APC	eBioscience™
GM-CSF	BVD2-21C11	PE-Dazzle	BioLegend
HLA-DR	G46-6	PE-CF594, PerCP-Cy5.5	BD Biosciences
ICOS	DX29	BV711	BD Biosciences
IFN-γ	4SB3	BUV737	BD Biosciences
IL-17	N49-653	BV785	BD Biosciences
LAG-3	T47-530	BV510	BD Biosciences
Ki67	B56	PE-Cy7	BD Biosciences
PD-1	EH12.1	BUV737	BD Biosciences
PD-L1	MH1	BV785	BD Biosciences
PD-L2	24F-10C12	PE/Dazzle	BioLegend
TIGIT	MBSA43	PE	eBioscience™
Tim-3	7D3	BV650	BD Biosciences
TNF	MAB11	BV650	BD Biosciences
